# Tobacco Craving, Nicotine Dependence, and Quit Intentions among LGB and Non-LGB High School Students: A Quasi-Experimental Analysis

**DOI:** 10.3390/ijerph18179000

**Published:** 2021-08-26

**Authors:** Sunday Azagba, Lingpeng Shan

**Affiliations:** 1 Ross and Carol Nese College of Nursing, The Pennsylvania State University, University Park, PA 16802, USA; 2 Division of Biostatistics, College of Public Health, The Ohio State University, Columbus, OH 43210, USA; shan.90@osu.edu

**Keywords:** tobacco craving, nicotine dependence, quit intention, LGB, health disparities

## Abstract

There is evidence of higher tobacco use among lesbian or gay and bisexual (LGB) populations. However, a limited number of studies have examined whether there are differences in potential indicators of future tobacco cessation behaviors between LGB and non-LGB populations. This study examined whether sexual identity is associated with craving, nicotine dependence, and quit intentions among high school students. Data were drawn from the 2020 National Youth Tobacco Survey (*n* = 1642). A propensity score matching (PSM) technique was used to address covariate imbalance among sexual identity groups. Additionally, subgroup analyses were performed for both males and females. The PSM results showed higher odds of craving among students who were gay or lesbian (aOR, 1.70; 95% CI = 1.13–2.55) and bisexual (aOR, 1.89; 95% CI = 1.23–2.92) compared to heterosexual (straight) students. In the sex-based subgroup analyses, we found that gay or lesbian (aOR, 1.92; 95% CI = 1.10–3.34) and bisexual (aOR, 3.12; 95% CI, 1.46–6.66) male students had significantly higher odds of craving when compared to heterosexual/straight male adolescents. However, the association was not significant in female students. Additionally, female bisexuals had significantly lower odds for quit intention (aOR, 0.48; 95% CI = 0.29–0.81) when compared to heterosexual/straight female adolescents. Results also showed no significant differences between LGB and non-LGB students for nicotine dependence. Sexual minority adolescents, especially male adolescents, were more likely to have tobacco cravings and bisexual females had lower odds of quit intention than heterosexual peers. Prevention efforts targeting this subpopulation may be beneficial.

## 1. Introduction

Despite years of progress, tobacco use is still the leading cause of preventable death in the United States, responsible for more than 480,000 deaths per year [1]. It was estimated that the prevalence of cigarette smoking among U.S. high school students decreased from 15.8% in 2011 to 4.6% in 2020 [2,3]. However, the increased popularity of vaping tobacco products among adolescents and youth poses significant public health concerns. E-cigarettes are now the most popular tobacco product used among U.S. middle and high school students. Approximately 20% of high school students reported past-month use of e-cigarettes [2,3,4]. The use of any form of tobacco product is unsafe for teens, which could cause addiction and damage to adolescent brain development [5].

Some populations suffer from disproportionately higher rates of tobacco product use [3,6,7,8,9]. Sexual minority students, particularly bisexual girls, had higher odds of tobacco product use, including cigarettes, cigars, smokeless tobacco, and e-cigarettes [6,8]. Moreover, sexual minorities initiated smoking and transitioned to daily smoking earlier than their straight peers [9]. Evidence suggests that sexual minorities might experience unique risk factors for tobacco use resulting from discrimination, stigma, violence, and social rejection, in addition to the common factors that the general population encounters [10,11,12,13,14,15].

A limited number of studies have examined differences in potential indicators of future tobacco cessation behaviors between LGB and non-LGB [16]. Prior research on LGB adolescents has mainly used local-level data and convenience samples [11,17,18,19]. Notably, not much is known currently, given that the tobacco product landscape has undergone significant changes in the past decade [17,18]. For example, using data from the 1996–2005 Growing Up Today Study (GUTS), Corliss and colleagues found that bisexual and gay/lesbian females had significantly higher nicotine dependence measures compared to completely heterosexual females [17]. Similarly, a 1999 GUTS found a significantly higher nicotine dependence score for gay/bisexuals girls and a lower score for boys than their heterosexual counterparts [18]. A study in Minneapolis and St. Paul metropolitan areas found that LGBT youth indicated a lower intention to quit cigarette smoking than non-LGBT youth [11]. These studies combined sexual minority subgroups such as gay and bisexual into one group [11,19], potentially not capturing essential differences among subgroups [17,18], making it difficult to compare studies. Furthermore, existing studies only examined cigarette-related cessation, potentially not capturing the popularity of emerging tobacco products. Lastly, limited prior studies used a rigorous analytical design that addressed potential imbalances between sexual identity subgroups. This study examines the differences in craving, nicotine dependence, and quit intention between LGB and non-LGB students using a nationally representative sample of high school students in the U.S. We used a propensity score matching (PSM) approach to address a potential imbalance in observable characteristics between sexual identity groups.

## 2. Methods

### 2.1. Data

**Figure 1:**
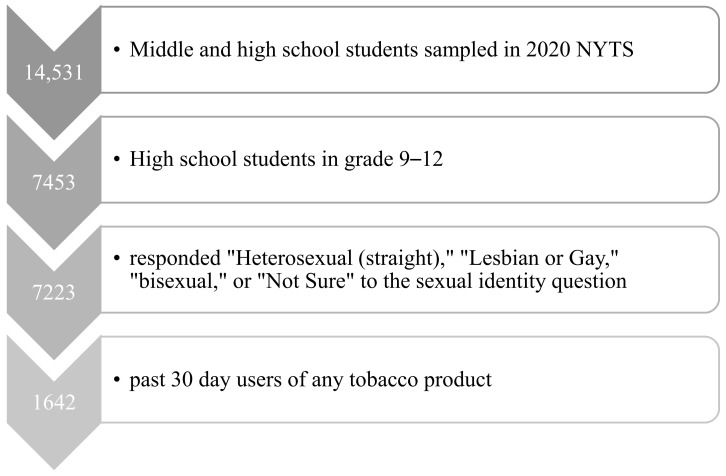
Flowchart of sample selection.

The present study is based on data from the 2020 National Youth Tobacco Surveys (NYTS). The NYTS is a cross-sectional survey of middle and high school students attending public and private schools in the United States. In addition to students’ demographic characteristics, the survey primarily collects information on tobacco-related beliefs, attitudes, behaviors, and exposure to tobacco-related advertising. The sample design was a three stage stratified cluster sample of randomly selected classes drawn from schools within the primary sampling units [20]. Classes were based on school course schedules to prevent students from having more than one chance of selection. Historically, NYTS has been conducted via paper and pencil questionnaires, and the NYTS began using electronic data collection methods starting in 2019. Participants were provided with a tablet to complete the survey, and their responses were anonymous, with approximately 35–45 min allotted to complete the survey. Those absent on the survey day could participate in make-up surveys using a web-based type automated similar to the tablet-based system. Participation in the NYTS was voluntary at both the school and student levels. Parents were allowed to opt their students out of participating in the survey, with consent obtained in one of two ways depending on the school, either passive or active permission. The 2020 survey cycle ended early due to the COVID-19 pandemic. The final sample consisted of 361 schools, of which 180 participated before school closures, with school participation of 49.9% and 87.4% of students completed questionnaires. The overall participation rate was 43.6% (including the school-level and student-level participation rates). The survey applied a weighting factor to each student record to adjust for nonresponse and varying selection probabilities. The final analytic sample consisted of 1642 high school students in grades 9–12 who were past 30 day users of any tobacco product and responded “Heterosexual(straight),” “Lesbian or Gay,” “bisexual,” or “Not Sure” to the sexual identity question (Figure 1).

### 2.2. Measures

The outcome variables of interest included tobacco craving, nicotine dependence, and intention to quit tobacco use. Craving was measured from the question, “During the past 30 days, have you had a strong craving or felt like you really needed to use a tobacco product of any kind?” Nicotine dependence was measured from the survey question “How soon after you wake up do you want to use a tobacco product?” Nicotine dependence was defined as using a tobacco product within 5 min after waking up. This measure is highly predictive of symptoms of dependence [21,22,23] and has shown reliability [24,25] in predicting nicotine dependence outcomes. The alternative measure of nicotine dependence was defined as using a tobacco product within 30 min after waking up. This measure was included in the Fagerstrom Tolerance Questionnaire, a widely used test of nicotine dependence [26]. The measure has also been shown to be appropriate and valid for adolescent smokers [27]. Quit intention was measured from the survey question “Are you seriously thinking about quitting the use of all tobacco products?” Two measures of quit intention were defined from the answers “yes, within the next 30 days” or “yes, within the next 6 months.”

Independent variables: sexual identity was measured from the question, “which of the following best describes you?” The possible responses included “Heterosexual (straight),” “Lesbian or Gay,” “bisexual,” and “Not Sure.” Other independent variables include demographic characteristics grade, sex (male and female), race/ethnicity (non-Hispanic White, non-Hispanic Black, Hispanic, and non-Hispanic other), tobacco use by household members (yes and no), exposure to tobacco marketing (ranging from zero to nine, detailed description can be found elsewhere [16]), and tobacco use status (dual-cigarette-and-e-cigarette-user, cigarette-only-smoker, e-cigarette-only-user, other-user).

### 2.3. Statistical Analysis

The sample demographic characteristics were examined for each sexual identity group (heterosexual, gay or lesbian, bisexual, and not sure). We reported the means and percentages for continuous and categorical variables, respectively. Descriptive statistics tests were performed to compare the statistically significant differences in characteristics among the four groups.

In order to reduce the cofounding effects in this observation study, differences in observed characteristics among four sexual identity groups (heterosexual, gay or lesbian, bisexual, and not sure) were adjusted using the propensity score weighting method. Specifically, propensity score weights were estimated using a generalized boosted model, which showed better performance in bias reduction and mean squared error than parametric approaches [28,29,30]. The estimation model adjusted for grade, sex, race/ethnicity, tobacco marketing exposure, household members’ tobacco use, and tobacco use status. Overlap assumption and imbalance tests were also conducted to check the PSM performance. Overlap assumption was checked using boxplots to compare the propensity scores’ distribution, whereas the imbalance test was performed by comparing absolute standardized mean differences (ASMD) [28]. After deriving weights from the PSM analysis, survey logistic regression was used to examine the association between sexual identity and the outcome variables. Separate subgroup analyses were performed for male and female students. Sampling weights were used in propensity score weights’ estimation, and for the outcome analysis, sampling weights were multiplied by the propensity score weights to derive the final weights [31,32]. All analyses were performed using SAS 9.4 (SAS Institute, Inc., Cary, NC, USA).

## 3. Results

Table 1 reports the sample characteristics and outcome variables by sexual identity. Bisexual high school students were more likely to be female (82.7%) than heterosexual peers (44.2%). The grade levels were significantly different by sexual identity groups. Gay or lesbian identity groups had the highest grade 9 (23.3%) compared to other sexual identity groups. No significant difference was observed for exposure to household members tobacco use by sexual identity. Among the 1642 students included in the study, more students (81.0%) identified as heterosexual/straight, 4.7% were gay or lesbian, 10.1% were bisexual, and 4.2% were unsure about their sexual identity.

**Figure 2:**
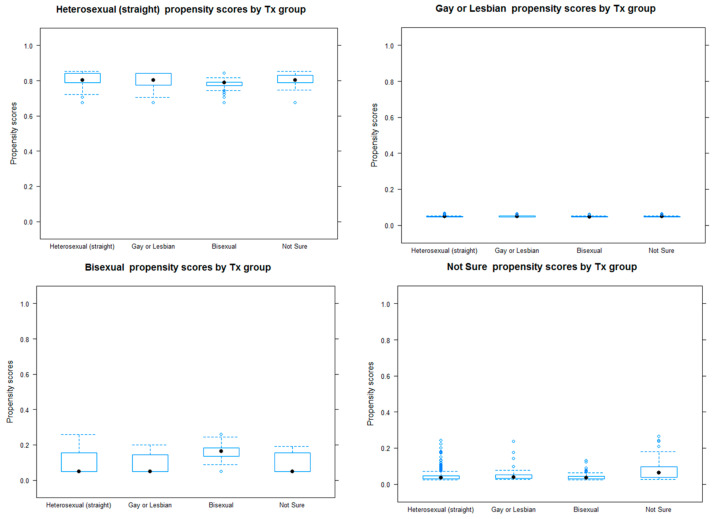
Graphical assessment of overlap assumption using boxplots of the estimated propensity distribution scores by sexual identity.

Table 2 reports the ASMD between the sexual identity groups on the observed characteristics before and after weighting, and the ASMD significantly decreased for the observable characteristics. Likewise, the propensity score distributions show a balance between groups after weighting (thereby satisfying the overlap assumption, see Figure 2).

Table 3 presents the outcomes regression results of craving, nicotine dependence, and intention to quit tobacco use on sexual identity status. Higher odds of craving were found among students who were gay or lesbian (aOR, 1.70; 95% CI = 1.13–2.55) and bisexual (aOR, 1.89; 95% CI = 1.23–2.92) compared to heterosexual (straight) students. More than half of heterosexual students reported that they had seriously thought about quitting the use of all tobacco products within the next 6 months (53.9%), while the percentage was less than 40% among sexual minority students (39.1% for gay or lesbian, 38.3% for bisexual, and 39.0% for not sure, Table 1). However, the association was not significant after using PSM to address the imbalance between groups. No significant association was found between sexual identity status and measures of nicotine dependence and quit intention.

In the sex-based subgroup analyses (Table 4), we found that gay or lesbian (aOR, 1.92; 95% CI, 1.10–3.34) and bisexual (aOR, 3.12; 95% CI = 1.46–6.66) male students had significantly higher odds of craving when compared to heterosexual/straight male adolescents. In contrast, the association was not significant in female students. Female bisexuals had significantly lower odds for quit intention within the next 6 months (OR, 0.48; 95% CI = 0.29–0.81) when compared to heterosexual/straight female adolescents. Results showed no significant association between sexual identity status and measures of nicotine dependence.

## 4. Discussion

The current study examined differences in craving, nicotine dependence, and quit intention between LGB and non-LGB using data from a nationally representative survey of high school students. A rigorous PSM rigorous approach was used to address an imbalance in characteristics between the sexual identity groups (heterosexual, gay or lesbian, bisexual, and not sure). We found that of the four sexual identity groups, bisexual and gay or lesbian adolescents had significantly higher odds of craving. Based on the incentive-sensitization model proposed by Robinson and Berridge, craving is one of the irresistible factors leading to relapse and subsequent tobacco use [33]. Thus, these findings complement the existing literature on the elevated risk of tobacco use among LGB subgroups [6,8,9,17,18,34].

Results showed substantial differences in a sex-based subgroup analysis with gay and bisexual males experiencing higher odds of craving. In contrast, previous studies identified tobacco use disparities among sexual minority females, especially bisexual females, whereas tobacco use behaviors were similar between sexual minorities and straight males [8,18,35]. Our results also showed that females bisexual students reported significantly lower odds of quit intention. Thus, the current study supports that bisexual minorities, especially bisexual females, potentially face an increased risk of persistent tobacco use [10,17,36,37]. The minority stress model documented that discrimination, stigma, violence, and social rejection could increase heightened stress levels and ultimately lead to risky health behaviors among sexual minorities [13]. Studies have also shown that bisexual individuals lacked social support from the minority community [38,39]. Future studies, including qualitative studies, could address the unique challenges of bisexual females.

In contrast to prior studies showing that sexual minorities experienced significantly higher nicotine dependence in both adolescents and adults [17,18,35], our no significant findings in nicotine dependence between sexual minorities and straight peers are also worth noting. One explanation could be the impact of recent tobacco prevention and control strategies, including public education campaigns, such as CDC’s Tips From Former Smokers campaign [40]. Promising impacts of such efforts have been shown in quit intention, quit attempts, and the risk of initiation among some subpopulations [41]. Additionally, though the structural anti-LGBT stigma remains, the recent shift in political and social changes in the rights and protections of LGBT people might have reduced the stress caused by structural stigmatization and discrimination [13,42]. It remains unknown to what extent the current environment may have attenuated the association between sexual identity and nicotine dependence.

The current study has some limitations that should be considered along with the findings reported. Typical of any school survey, the NYTS may not be generalizable to youth not attending schools (e.g., those home schooling or who have dropped out of school). The NYTS is a self-reported survey, which could be subject to recall bias. Additionally, the “Not sure” response could represent a broader group. For example, it could represent individuals who are unsure about their sexual identity, those who do not fully understand the sexual identity question, and other subgroups (e.g., transgender). Finally, the 2020 NYTS had lower participation rates due to the COVID-19 pandemic. However, the survey weight adjustments adjusted for nonresponse to reduce potential bias [3,20].

## 5. Conclusions

This study found that bisexual and gay or lesbian adolescents, especially male adolescents, had significantly higher odds of craving. Bisexual females had lower odds of quit intention compared to their heterosexual peers. While previous studies have shown that sexual minorities experienced significantly higher nicotine dependence in both adolescents and adults [17,18,35], this study found no significant differences between LGB and non-LGB students. It remains unknown to what extent the recent shift in political and social changes in the rights and protections of sexual minority populations might have attenuated the association between sexual identity and nicotine dependence.

## Figures and Tables

**Table 1:** Summary statistics of study participants, 2020 NYTS.

	General Population	Heterosexual (Straight)	Gay or Lesbian	Bisexual	Not Sure	*p*-Value
N	1642	1323	79	168	72	
Grade						**<0.01**
9th	18.39 (15.21, 21.57)	18.30 (14.89, 21.72)	23.26 (11.43, 35.09)	19.65 (11.81, 27.48)	11.61 (3.42, 19.80)	
10th	24.56 (21.72, 27.40)	24.08 (21.11, 27.05)	28.56 (14.04, 43.07)	19.95 (12.95, 26.94)	40.37 (26.74, 54.00)	
11th	26.13 (23.63, 28.62)	25.62 (22.71, 28.52)	21.07 (11.15, 31.00)	27.70 (21.21, 34.19)	37.92 (28.14, 47.70)	
12th	30.92 (27.83, 34.01)	32.00 (28.68, 35.33)	27.11 (15.76, 38.46)	32.71 (24.48, 40.94)	10.09 (2.35, 17.84)	
Sex						**<0.0001**
Male	52.32 (48.78, 55.87)	55.84 (51.83, 59.85)	59.91 (46.23, 73.59)	17.28 (10.16, 24.40)	59.90 (46.93, 72.86)	
Female	47.68 (44.13, 51.22)	44.16 (40.15, 48.17)	40.09 (26.41, 53.77)	82.72 (75.60, 89.84)	40.10 (27.14, 53.07)	
Race/ethnicity						0.24
Non-Hispanic White	56.26 (50.34, 62.18)	57.54 (50.90, 64.18)	49.06 (31.91, 66.22)	50.54 (40.67, 60.41)	53.56 (40.01, 67.12)	
Non-Hispanic Black	8.10 (5.22, 10.97)	7.43 (4.49, 10.36)	14.96 (4.14, 25.78)	7.59 (1.71, 13.47)	14.47 (4.33, 24.60)	
Hispanic	25.05 (18.87, 31.23)	23.91 (17.29, 30.53)	26.99 (13.77, 40.20)	33.28 (24.75, 41.81)	24.78 (12.16, 37.39)	
Non-Hispanic Other	10.60 (7.98, 13.22)	11.12 (7.82, 14.43)	8.99(1.86, 16.13)	8.59(3.26, 13.92)	7.20 (0.00, 14.43)	
Exposure to tobacco marketing	3.76 (3.54, 3.99)	3.75 (3.51, 3.98)	4.05 (2.84, 5.27)	3.71 (3.19, 4.24)	3.97 (3.11, 4.84)	0.91
Tobacco use by household members						0.27
Yes	51.85 (48.55, 55.15)	50.89 (46.81, 54.96)	49.40 (36.45, 62.35)	61.41 (50.49, 72.32)	49.71 (34.14, 65.28)	
No	48.15 (44.85, 51.45)	49.11 (45.04, 53.19)	50.60 (37.65, 63.55)	38.59 (27.68, 49.51)	50.29 (34.72, 65.86)	
Tobacco use status						**<0.001**
Dual-cigarette-and-e-cigarette-user	17.58 (13.80, 21.36)	15.41 (11.24, 19.59)	21.22 (10.89, 31.54)	26.67 (19.24, 34.10)	33.38 (20.02, 46.74)	
Cigarette-only-smoker	1.73 (1.05, 2.40)	1.81 (1.03, 2.60)	0.93 (0.00, 2.49)	0.86 (0.00, 2.20)	3.09 (0.00, 6.96)	
E-cigarette-only-user	73.15 (69.51, 76.78)	75.77 (71.84, 79.69)	71.94 (60.52, 83.35)	59.00 (49.96, 68.04)	58.06 (45.64, 70.49)	
Other-user	7.55 (5.53, 9.56)	7.01 (5.33, 8.69)	5.91 (0.00, 11.99)	13.47 (5.62, 21.33)	5.46 (0.21, 10.71)	
Craving						**0.04**
Yes	34.18 (30.80, 37.56)	32.57 (29.28, 35.86)	45.17 (34.60, 55.73)	42.01 (30.63, 53.39)	34.14 (22.10, 46.18)	
No	65.82 (62.44, 69.20)	67.43 (64.14, 70.72)	54.83 (44.27, 65.40)	57.99 (46.61, 69.37)	65.86 (53.82, 77.90)	
Nicotine Dependence within 5 min						0.80
Yes	11.85 (10.12, 13.57)	11.44 (9.47, 13.41)	15.71 (6.06, 25.36)	12.92 (6.34, 19.49)	12.73 (2.37, 23.10)	
No	88.15 (86.43, 89.88)	88.56 (86.59, 90.53)	84.29 (74.64, 93.94)	87.08 (80.51, 93.66)	87.27 (76.90, 97.63)	
Nicotine Dependence within 30 min						0.91
Yes	19.44 (17.11, 21.77)	19.11 (16.86, 21.37)	22.54 (12.60, 32.48)	20.57 (11.73, 29.41)	19.51 (8.80, 30.22)	
No	80.56 (78.23, 82.89)	80.89 (78.63, 83.14)	77.46 (67.52, 87.40)	79.43 (70.59, 88.27)	80.49 (69.78, 91.20)	
Quit intention within next 30 days						0.13
Yes	35.29 (31.93, 38.65)	37.05 (33.62, 40.49)	30.52 (17.47, 43.57)	27.41 (18.40, 36.43)	26.55 (11.74, 41.35)	
No	64.71 (61.35, 68.07)	62.95 (59.51, 66.38)	69.48 (56.43, 82.53)	72.59 (63.57, 81.60)	73.45 (58.65, 88.26)	
Quit intention within next 6 months						**<0.01**
Yes	50.96 (47.44, 54.49)	53.90 (50.51, 57.28)	39.14 (24.40, 53.88)	38.28 (27.88, 48.68)	38.96 (24.81, 53.10)	
No	49.04 (45.51, 52.56)	46.10 (42.72, 49.49)	60.86 (46.12, 75.60)	61.72 (51.32, 72.12)	61.04 (46.90, 75.19)	

The sample was restricted to 1642 high school students in grades 9–12 who were past 30 day users of any tobacco product and responded “Heterosexual(straight),” “Lesbian or Gay,” “bisexual,” or “Not Sure” to the sexual identity question. Categorical characteristics are presented in weighted column percentages (95% confidence interval) while continuous characteristic, exposure to tobacco marketing, is presented in weighted mean (95% confidence interval). Rao-Scott chi-square tests and ANOVA tests were used to detect significant differences among groups. Significant *p*-values (*p* < 0.05) were presented in boldface.

**Table 2:** Pre- and post-propensity weighted means for covariates and absolute standardized mean difference (ASMD).

	Propensity Score Mean (Unweighted) (ASMD from Heterosexual (Straight))				Propensity Score Mean (Weighted) (ASMD from Heterosexual (Straight))			
	Heterosexual (Straight)	Gay or Lesbian	Bisexual	Not Sure	Heterosexual (Straight)	Gay or Lesbian	Bisexual	Not Sure
Grade	0.32	0.28 (0.10)	0.29 (0.08)	0.18 (0.31)	0.32	0.28 (0.10)	0.33 (0.02)	0.23 (0.20)
Sex	0.54	0.58 (0.08)	0.17 (0.75)	0.60 (0.11)	0.52	0.58 (0.12)	0.48 (0.08)	0.48 (0.09)
Race/ethnicity	0.58	0.48 (0.21)	0.55 (0.06)	0.43 (0.31)	0.58	0.49 (0.19)	0.56 (0.04)	0.57 (0.01)
Exposure to tobacco marketing	3.82	2.73 (0.43)	3.33 (0.19)	3.00 (0.33)	3.83	2.88 (0.38)	3.07 (0.30)	3.35 (0.19)
Tobacco use by household members	0.51	0.53 (0.04)	0.64 (0.27)	0.50 (0.02)	0.51	0.52 (0.01)	0.61 (0.20)	0.58 (0.13)
Tobacco use status	0.77	0.67 (0.23)	0.64 (0.29)	0.63 (0.33)	0.77	0.67 (0.20)	0.66 (0.24)	0.71 (0.11)

ASMDs are between heterosexual (straight) and other sexual minority groups.

**Table 3:** Logistic regression of craving, nicotine dependence, and intention to quit tobacco use on sexual identity status.

	Gay or Lesbian	Bisexual	Not Sure	Heterosexual (Straight)
Craving	**1.70 (1.13, 2.55)**	**1.89 (1.23, 2.92)**	1.12 (0.57, 2.19)	Ref
Nicotine Dependence within 5 min	1.49 (0.68, 3.26)	1.38 (0.67, 2.84)	0.99 (0.33, 2.96)	Ref
Nicotine Dependence within 30 min	1.15 (0.62, 2.13)	1.40 (0.80, 2.45)	1.11 (0.50, 2.46)	Ref
Quit intention within next 30 days	0.76 (0.42, 1.39)	0.63 (0.35, 1.16)	0.51 (0.20, 1.32)	Ref
Quit intention within next 6 months	0.58 (0.31, 1.06)	0.59 (0.34, 1.03)	0.57 (0.28, 1.14)	Ref

Ref represents the reference category, and significant odds ratios (*p* < 0.05) were presented in boldface.

**Table 4:** Logistic regression of craving, nicotine dependence, and intention to quit tobacco use on sexual identity status, by sex.

	Male				Female			
	Gay or Lesbian	Bisexual	Not Sure	Heterosexual (Straight)	Gay or Lesbian	Bisexual	Not Sure	Heterosexual (Straight)
Craving	**1.92 (1.10, 3.34)**	**3.12 (1.46, 6.66)**	1.30 (0.51, 3.31)	Ref	1.46 (0.68, 3.14)	1.19 (0.67, 2.10)	0.93 (0.28, 3.12)	Ref
Nicotine Dependence within 5 min	1.73 (0.51, 5.89)	2.11 (0.74, 6.03)	1.39 (0.45, 4.28)	Ref	1.26 (0.45, 3.48)	0.87 (0.43, 1.78)	0.64 (0.07, 5.54)	Ref
Nicotine Dependence within 30 min	1.05 (0.40, 2.77)	2.13 (0.95, 4.74)	1.47 (0.65, 3.32)	Ref	1.31 (0.54, 3.14)	0.88 (0.46, 1.68)	0.75 (0.18, 3.13)	Ref
Quit intention within next 30 days	0.88 (0.42, 1.86)	0.71 (0.24, 2.07)	0.63 (0.19, 2.02)	Ref	0.62 (0.23, 1.63)	0.57 (0.32, 1.00)	0.39 (0.13, 1.24)	Ref
Quit intention within next 6 months	0.61 (0.31, 1.21)	0.74 (0.27, 2.04)	0.76 (0.32, 1.80)	Ref	0.51 (0.18, 1.44)	**0.48 (0.29, 0.81)**	0.38 (0.14, 1.03)	Ref

Ref represents the reference category, and significant odds ratios (*p* < 0.05) were presented in boldface.

## Data Availability

Publicly available datasets.
